# Is rate–pressure product of any use in the isolated rat heart? Assessing cardiac ‘effort’ and oxygen consumption in the Langendorff‐perfused heart

**DOI:** 10.1113/EP085380

**Published:** 2015-12-16

**Authors:** Dunja Aksentijević, Hannah R. Lewis, Michael J. Shattock

**Affiliations:** ^1^ British Heart Foundation Centre of Research ExcellenceKing's College LondonThe Rayne Institute, St Thomas’ HospitalLondonUK

## Abstract

**New Findings:**

- **What is the central question of this study?**
  Rate–pressure product (RPP) is commonly used as an index of cardiac ‘effort’. In canine and human hearts (which have a positive force–frequency relationship), RPP is linearly correlated with oxygen consumption and has therefore been widely adopted as a species‐independent index of cardiac work. However, given that isolated rodent hearts demonstrate a negative force–frequency relationship, its use in this model requires validation.
- **What is the main finding and its importance?**
  Despite its widespread use, RPP is not correlated with oxygen consumption (or cardiac ‘effort’) in the Langendorff‐perfused isolated rat heart. This lack of correlation was also evident when perfusions included a range of metabolic substrates, insulin or β‐adrenoceptor stimulation.

Langendorff perfusion of hearts isolated from rats and mice has been used extensively for physiological, pharmacological and biochemical studies. The ability to phenotype these hearts reliably is, therefore, essential. One of the commonly used indices of function is rate–pressure product (RPP); a rather ill‐defined index of ‘work’ or, more correctly, ‘effort’. Rate–pressure product, as originally described in dog or human hearts, was shown to be correlated with myocardial oxygen consumption ($M{\dot{V}}_{O_{2}}$). Despite its widespread use, the application of this index to rat or mouse hearts (which, unlike the dog or human, have a negative force–frequency relationship) has not been characterized. The aim of this study was to examine the relationship between RPP and $M{\dot{V}}_{O_{2}}$ in Langendorff‐perfused rat hearts. Paced hearts (300–750 beats min^−1^) were perfused either with Krebs–Henseleit (KH) buffer (11 mm glucose) or with buffer supplemented with metabolic substrates and insulin. The arteriovenous oxygen consumption ($M{\dot{V}}_{O_{2}}$) was recorded. Metabolic status was assessed using ^31^P magnetic resonance spectroscopy and lactate efflux. Experiments were repeated in the presence of isoprenaline and in unpaced hearts where heart rate was increased by cumulative isoprenaline challenge. In KH buffer‐perfused hearts, $M{\dot{V}}_{O_{2}}$ increased with increasing heart rate, but given that left ventricular developed pressure decreased with increases in rate, RPP was not correlated with $M{\dot{V}}_{O_{2}}$, lactate production or phosphocreatine/ATP ratio. Although the provision of substrates or β‐adrenoceptor stimulation changed the shape of the RPP–$M{\dot{V}}_{O_{2}}$ relationship, neither intervention resulted in a positive correlation between RPP and oxygen consumption. Rate–pressure product is therefore an unreliable index of oxygen consumption or ‘cardiac effort’ in the isolated rat heart.

## Introduction

Langendorff perfusion of hearts isolated from rats and mice has been used extensively for physiological, pharmacological, biochemical and molecular studies for many years. The ability to modify mice genetically has further enhanced the utility of this technique and the ability to phenotype these hearts reliably has become essential. One of the commonly used indices of function is rate–pressure product (RPP), which is frequently adopted as some rather ill‐defined index of ‘work’ or, more correctly, ‘effort’. Rate–pressure product was originally described by Katz & Feinberg (1958) as an index of ‘cardiac effort’ in dog hearts and shown to be linearly correlated with oxygen consumption. This correlation with oxygen consumption was confirmed in human hearts by Kitamura *et al*. (1972). However, despite its widespread use, the application of this index to rat and mouse hearts, has a problem; namely, whereas dogs, humans and most other large mammals show a positive force–frequency relationship (FFR) (Bers, 2000) (that is, as heart rate increases so does force development), this relationship is flat or even negative in the electrically paced isolated rat or mouse heart (Sutherland *et al*. 2003). Thus, although RPP may increase with heart rate and be correlated with myocardial O_2_ consumption ($M{\dot{V}}_{O_{2}}$) in the human heart, what happens in the isolated rat or mouse heart has never been defined.

This relationship is further complicated by the fact that the heart *in vivo* rarely, if ever, encounters a substantial rise in heart rate without β‐adrenoceptors being occupied. Despite this, many studies use electrical pacing to elevate rate in the absence of β‐agonists (Sutherland *et al*. 2003). β‐Adrenergic stimulation will phosphorylate key players in the pathway of excitation–contraction coupling, such as L‐type Ca^2+^ channels, phospholamban, phospholemman, troponin I and the ryanodine receptor; changes that will modulate contractility and hence oxygen consumption (Lindemann *et al*. 1983; Vassallo *et al*. 1994; Bers, 2000; Shattock, 2009). In this context, β‐adrenergic stimulation has been shown to transform the negative force–frequency relationship in the isolated mouse heart into a positive one (Kennington, 2009).

Not only does the isolated heart usually lack adrenergic tone, but it is frequently provided with only glucose (and no insulin) as a metabolic substrate. The lack of the normal range of substrate provision is again likely to influence the relationship between metabolic efficiency, myocardial oxygen consumption and contractility, particularly when heart rate is elevated.

The aim of this study was to examine the relationship between RPP and $M{\dot{V}}_{O_{2}}$ in the isolated Langendorff‐perfused rat heart and, in keeping with its original definition, to establish whether RPP is an appropriate index of cardiac effort. In order to mimic the effect of physiological conditions on the investigated RPP–$M{\dot{V}}_{O_{2}}$ relationship, in addition to experiments with the most commonly used crystalloid hyperglycaemic Krebs–Henseleit (KH) buffer, perfusions were carried out with KH buffer containing physiological concentrations of exogenous metabolic substrates and in the presence of β‐adrenergic stimulation.

## Methods

### Ethical approval

All experiments were approved by the institutional ethical review committee and conform to the UK Animals (Scientific Procedures) Act 1986 incorporating European Directive 2010/63/EU.

### Animals

Male Wistar rats (316 ± 58 g body weight, *n* = 36) were purchased from Harlan Laboratories (UK). Rats were housed in pathogen‐free cages with a 12 h–12 h light–dark cycle, controlled humidity and temperature (20–22°C) and fed standard rat chow and water *ad libitum*.

### Isolated heart perfusion

Rats were anaesthetized by i.p. injection of 0.9 ml sodium pentobarbitone 20% w/v (Pentoject, Animalcare Ltd., York, UK). Hearts (wet weight 1.31 ± 0.23 g, *n* = 36) were rapidly excised and arrested in ice‐cold KH buffer. The aorta was cannulated and secured with sutures (Mersilk 3‐0; Ethicon, Somerville, NJ, USA). Hearts were perfused at a constant perfusion pressure of 80 ± 2 mmHg using a peristaltic pump (Gilson Minipuls 4, Middleton, WI, USA) and a feedback control system (STH Pump Controller; AD Instruments, Oxford, UK). Buffer was equilibrated with 95% O_2_–5% CO_2_ using a custom‐made counter‐current membrane oxygenator consisting of spirally wound Silastic tubing (1.47 mm i.d., 1.96 mm o.d.; VWR International, Lutterworth, UK) continually flushed with gas at 37°C. A fluid‐filled balloon, attached to a pressure transducer, was inserted into the left ventricle and inflated to give an end‐diastolic pressure between 3 and 8 mmHg. Left ventricular pressure (LVP), perfusion pressure, coronary flow and arterial and venous oxygen tensions were recorded using a PowerLab recorder and LabChart 7.0 software (ADInstruments). Functional parameters were averaged for ∼80 cardiac cycles at 5 min intervals.

Left ventricular developed pressure (LVDP) was calculated as the difference between maximal systolic (SP) and end‐diastolic pressures. Rate–pressure product was defined as the product of heart rate (HR) and left ventricular developed pressure: RPP = (HR ×LVDP)/1000.

Venous effluent samples, taken during equilibration and within the last minute of each frequency increase, were immediately frozen in liquid nitrogen and stored at −80°C for analysis of lactate concentration. Samples were then thawed on ice before analysis in triplicate with a YSI 2300 Stat Plus Lactate Analyser (YSI Life Sciences, Tunbridge Wells, UK).

### Myocardial oxygen consumption

Arterial and venous oxygen was measured using two 420‐μm‐diameter fibre‐optic oxygen sensors (Ocean Optics, NeoFox, UK) calibrated prior to use with 0% O_2_ (using the reducing agent 4% w/v sodium hydrosulphite) and 100% O_2_ gassed water. One probe was placed in the aortic inflow arterial line and the second, venous probe was placed in the right ventricular outflow tract inserted via the pulmonary artery. The oxygen content of arterial and venous perfusate was recorded as percentage O_2_ saturation. Atmospheric pressure (in millimetres of mercury) was measured daily, and $M{\dot{V}}_{O_{2}}$ was then calculated according to the following equation:

MV˙O2=PB×Δ%O2100×SO2× CF  Dry  Wt  in  micromoles  per  minute  per  gram  dry  weight

Where *P*_B_ is atmospheric pressure (in millimetres of mercury), Δ%O_2_ is the arteriovenous difference in percentage O_2_ saturation, $S_{O_{2}}$ is the solubility of oxygen in Krebs‐Henseleit (KH) buffer = 1.22 ×10^−3^ μmol ml^−1^ mmHg^−1^ at BTSP (Chemnitius *et al*. 1985; Schenkman *et al*. 2003), CF is coronary flow (in millilitres per minute) and Dry Wt is the dry weight of the heart (in grams).

### Perfusion solutions

#### Krebs–Henseleit buffer

The standard KH buffer contained (mm): 118 NaCl, 25 NaHCO_3_, 4.8 KCl, 1.2 KH_2_PO4, 1.2 MgSO_4_.7H_2_O, 1.4 CaCl_2_.7H_2_O and 11 glucose, pH 7.4.

#### Substrate‐enriched metabolic (KH_metab_) buffer

In some experiments, the glucose concentration of the KH buffer was 5 mmol l^−1^ and the additional substrates and compounds included the following (mm): 1 sodium l‐lactate, 0.1 sodium pyruvate, 0.5 l‐glutamic acid monosodium salt monohydrate; 5 mU l^−1^ insulin (NovoRapid insulin; Novo Nordisk, Denmark) and 0.3 sodium palmitate with 3% (w/v) bovine serum albumin (BSA; Proliant Biologicals, Ankeny, IA, USA; Smith *et al*. 2010). Prior to inclusion, BSA was prepared as follows.

#### Purification of BSA

Bovine serum albumin was dissolved (30% w/v) in a solution containing (mm): 118 NaCl and 2.5 CaCl_2_.7H_2_O. Visking dialysis tubing (Medicell Membranes Ltd., London, UK) was prepared by boiling for 10 min in a solution containing (mm): 1 EDTA, 119 NaHCO_3_, rinsed with ddH_2_O and then boiled for another 10 min in 1 mmol l^−1^ EDTA. The tubing was given a final rinse in ddH_2_O before the BSA solution was transferred and left to dialyse for 48 h at 4°C against a 20‐fold larger volume of (mm): 118 NaCl and 2.5 CaCl_2_.7H_2_O. This removed molecular impurities (molecular mass cut‐off of 12–14 kDa) and saturated the Ca^2+^‐binding sites of BSA (Smith *et al*. 2010).

All buffers were filtered through a 5.0 μm cellulose nitrate membrane filter (Whatman, Maidstone, UK). Appropriate corrections were made for Na^+^ and Ca^2+^ concentrations in the KH buffer to account for their presence in BSA solution stock (Smith *et al*. 2010).

### Perfusion protocols

Hearts were paced via epicardial silver wire electrodes placed at the apex of the left ventricle and the right atrium. After a 20 min functional equilibration period and continuous pacing at ∼300 beats min^−1^, the frequency of the pacing was increased every 5 min in 1 Hz increments up to 720 beats min^−1^. Hearts were divided into the following five groups: (i) crystalloid perfusion (KH buffer); (ii) perfusion using the substrate‐enriched KH_metab_ buffer; (iii) crystalloid perfusion plus β‐adrenergic receptor stimulation with isoprenaline hydrochloride (10 nm); (iv) KH_metab_ buffer plus β‐adrenergic receptor stimulation with isoprenaline hydrochloride (ISO; 10 nm); and (v) unpaced, crystalloid perfusion with stepwise logarithmic increases in ISO concentration (from 0.01 to 1000 nm). Sodium l‐ascorbate (0.05 mm) was added to both the vehicle and ISO‐containing buffer to prevent catecholamine oxidation. In group (iv), hearts were initially perfused with the vehicle buffer (containing ascorbate alone) for a 20 min functional equilibration and paced at 350 beats min^−1^ before switching to the ISO buffer. In these hearts, higher baseline pacing rates were used to account for the positive chronotropic effect of ISO. Hearts were perfused with the KH_metab_ buffer + ISO for a further 10 min equilibration to allow the function to stabilize before the pacing protocol was initiated and effluent samples were collected as described above.

### ^31^P Magnetic resonance spectroscopy (MRS)

^31^P Magnetic resonance spectroscopy experiments were conducted in a vertical‐bore Bruker Avance III 400 MHz wide‐bore spectrometer (Bruker Biospin, Switzerland) with triple axis gradients, a microimaging probe and an exchangeable 15 mm ^1^H/^31^P dual tune birdcage coil (m2m Imaging, Cleveland, OH, USA). Following cannulation on a specially constructed, MR‐compatible umbilical perfusion rig, the rat heart was lowered into the centre of the magnet at the position previously verified as the isocentre of the coil. Temperature calibration was performed *in situ* using a capillary containing ethylene glycol at the position of the heart. Shimming was carried out on the ^1^H signal of water, which was <50 Hz. Fully relaxed ^31^P spectra were acquired with an experiment duration of 13 min, 200 scans, repetition time of 3.85 s, a 90 deg flip angle, 9.8 kHz sweep width and acquisition data size of 16,384 points. After functional equilibration, hearts were perfused with the KH buffer and paced for 10 min at 350 beats min^−1^ followed by 10 min perfusion at 500 beats min^−1^. All spectra were processed with 20 Hz line broadening, and peak integrals were fitted using Bruker Top Spin version 2.1 software.

### Statistical analysis

The results are presented as means ± SEM. Statistical significance of functional parameters was assessed using GraphPad Prism 6 software (GraphPad Software Inc., La Jolla, CA, USA). Linear relationships were fitted by least‐squares analysis and correlation was assessed using Pearson's product–moment correlation coefficient (*r*). Some relationships appeared to have two linear phases either side of an inflection. In these cases, the whole data range was fitted initially using the entire data set and a single Pearson's *r* value derived. Subsequently, the two linear phases were then fitted separately with the inflection arbitrarily defined. If either of these two phases reported a higher *r* value than the whole data range, then this double‐fit is presented rather than a single linear regression. Some data were best described by a single exponential function according to *Y* = (*Y*_0_ − Plateau) × exp(−*K* × *X*) + Plateau. Differences between two means were analysed using Student's unpaired *t* test and considered significant when *P* < 0.05.

## Results

### Rate–pressure product and cardiac function in the KH‐perfused heart

**Figure 1:**
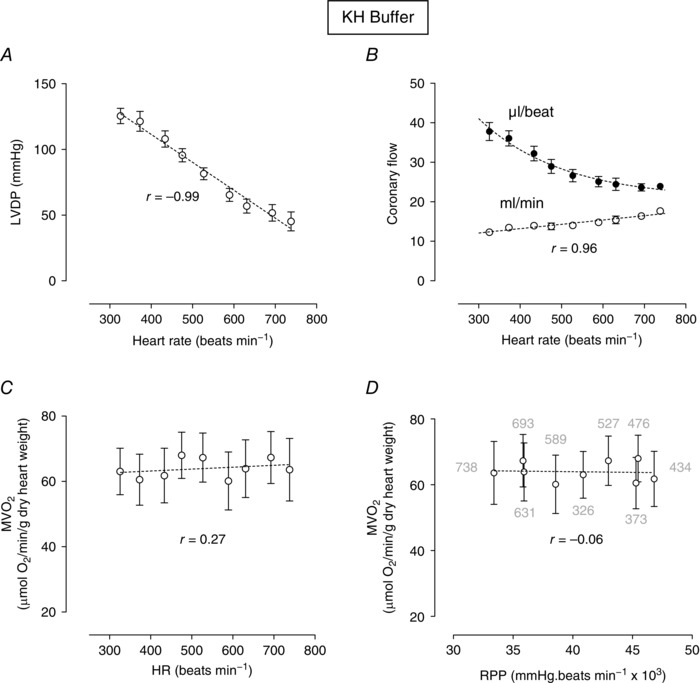
**Myocardial oxygen consumption** ($M{\dot{V}}_{O_{2}}$)**, left ventricular developed pressure (LVDP) and coronary flow plotted as a function of heart rate or rate–pressure product (RPP) in crystalloid Krebs–Henseleit (KH) buffer perfusions** Relationship between pacing rate and LVDP (*A*) and coronary flow (*B*). Myocardial oxygen consumption is plotted as a function of heart rate (*C*) or RPP (*D*). Data are means ± SEM (*n* = 8). The *r* value is the Pearson correlation for each linear relationship. Non‐linear fits are as described in the Methods.

Figure 1 *A* shows the relationship between LVDP and heart rate. There was a negative linear relationship between increasing heart rate and LVDP (a negative FFR). Increasing the pacing rate from 300 to 750 beats min^−1^ resulted in a 79% decrease in LVDP from 103 to 21 mmHg and a fivefold increase in left ventricular end‐diastolic pressure from baseline levels (from 4.9 ± 1.7 to 33 ± 5.1 mmHg; not shown). The slope of this negative LVDP–frequency relationship was −0.99 mmHg beat min^−1^. In this pacing protocol, there was a gradual increase in absolute coronary flow as heart rate increased (Fig. 1 *B*). Interestingly, despite the gradual vasodilatation in response to increasing heart rate, coronary flow expressed per beat declined with increasing rate (Fig. 1 *B*). Myocardial oxygen consumption as a function of heart rate and RPP are shown in Fig. 1 *C* and *D*. Figure 1 *C* shows that the combination of an increase in heart rate and the associated reduction in LVDP (Fig. 1 *A*) results in no net change in O_2_ consumption over a wide range in heart rates. Hence, unlike the positive correlation originally described by Katz & Feinberg (1958), it is clear that there was absolutely no correlation between RPP and myocardial oxygen consumption (*r* = 0.037; Fig. 1 *D*). The numbers next to the symbols in Fig. 1 *D* indicate the heart rate at each point. Interestingly, the absolute arteriovenous oxygen difference remained largely constant and within a narrow range [0.17–0.18 μmol (g dry wt)^−1^] at all pacing frequencies (see Table S1). This is in accord with Laurent *et al*. (1956), who concluded that net oxygen delivery to the myocardium is primarily determined by the prevailing coronary flow rather than any change in the efficiency of arteriovenous extraction.

### Impact of increasing heart rate on cardiac metabolism

**Figure 2:**
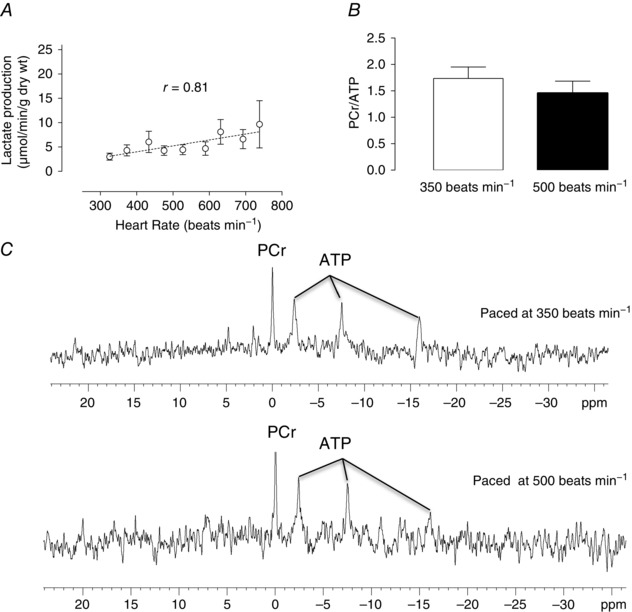
**Metabolic profile of crystalloid KH buffer‐perfused hearts** *A*, lactate production measured in coronary effluent at each pacing rate (*n* = 8). *B*, comparison of PCr/ATP ratios at physiological heart rates 350 (baseline) and 500 beats min^−1^ (*n* = 4). *C*, representative *in situ* perfused and paced heart ^31^P spectra. Abbreviations: ATP, adenosine triphosphate; and PCr, phosphocreatine. Averaged data are means ± SEM.

One of the many limitations of the isolated KH buffer‐perfused heart is the lack of oxygen‐carrying capacity of the perfusate. At high heart rates, it is possible that this may lead to a demand ischaemia. Figure 2 *A*, however, shows that, over the range of heart rates tested, there was no net increase in myocardial lactate production as might have been indicative of a gradually developing demand ischaemia. Likewise, ^31^P nucelar magnetic resonance spectra (Fig. 2 *C*), ATP and phosphocreatine (PCr) content, PCr/ATP ratios (Fig. 2 *B*) and intracellular pH were all unaffected by increasing the heart rate from 350 to 500 beats min^−1^ (a heart rate range equivalent to that measured *in vivo* in rats (Bolter & Atkinson, 1988).

### Provision of substrate‐enriched metabolic buffer

**Figure 3:**
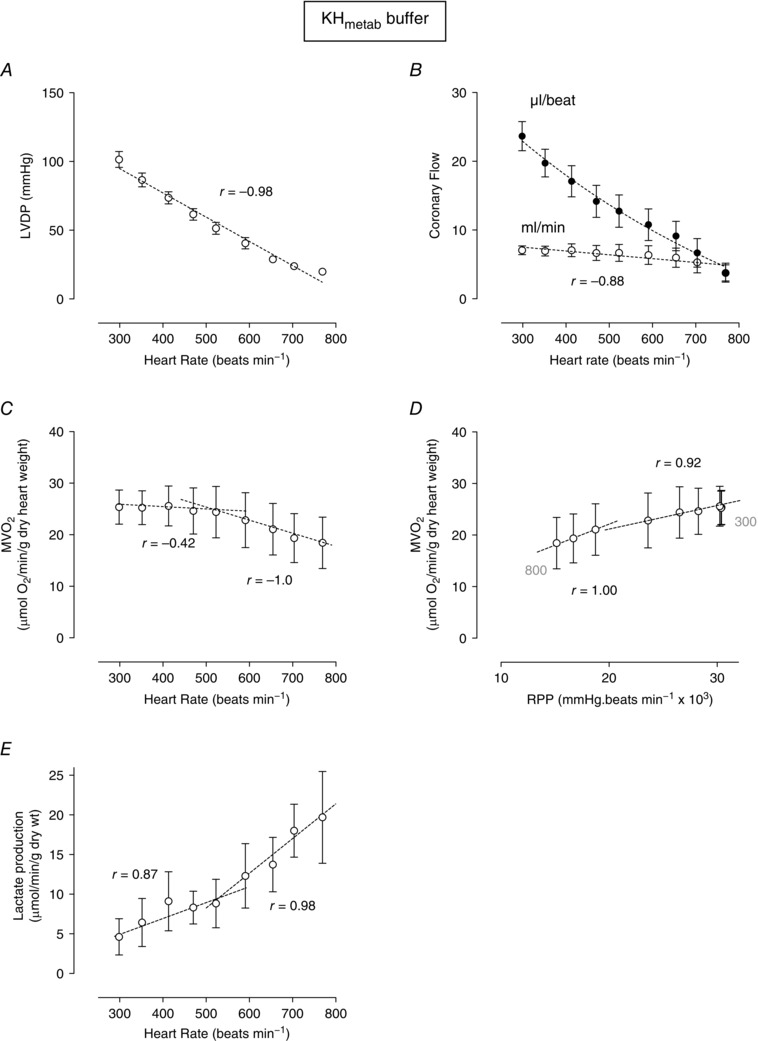
**Myocardial oxygen consumption, LVDP, coronary flow and lactate production plotted as a function of heart rate or RPP in buffer supplemented with metabolic substrates (KH_metab_)** Relationship between pacing rate and LVDP (*A*) and coronary flow (*B*). Myocardial oxygen consumption is plotted as a function of heart rate (*C*) or RPP (*D*). The lactate production measured in coronary effluent at each pacing rate is shown in *E*. Data are means ± SEM (*n* = 9). The *r* value is the Pearson correlation for each linear relationship, and inflections and regions of linear fit were defined as described in the Methods section.

Not only is the isolated heart potentially oxygen deficient at high heart rates but it may also be substrate deficient, being typically provided only with glucose (and no insulin) as a metabolic substrate. In order to investigate whether this impacts upon metabolic efficiency and the relationship between RPP and oxygen consumption, the protocols in Fig. 1 were repeated with a substrate‐enriched metabolic buffer (KH_metab_). Figure 3 *A* shows that, as in KH buffer, the LVDP–frequency relationship remained negative (slope −0.98 mmHg beat min^−1^). In contrast, however, baseline coronary flow was reduced at 325 beats min^−1^ in KH_metab_ buffer to 7.1 ± 0.6 ml min^−1^ (cf. 12.3 ± 0.7 ml min^−1^ in KH buffer at 350 beats min^−1^; Fig. 3 *B*). With increasing heart rate, absolute coronary flow (in millilitres per minute) remained relatively constant over the range 325–650 beats min^−1^, declining slightly over the entire frequency range (*r* = −0.88). As in KH perfusion, coronary flow per beat declined with increasing frequency (Fig. 3 *B*). The relationship between heart rate and absolute coronary flow was mirrored by a similar profile of oxygen consumption (Fig. 3 *C*). In these hearts, at baseline (325 beats min^−1^) the $M{\dot{V}}_{O_{2}}$ was significantly lower in KH_metab_‐perfused hearts compared with KH‐perfused hearts [$M{\dot{V}}_{O_{2}}$ in KH_metab_ = 0.93 ± 0.1 *versus* 2.27 ± 0.2 μmol O_2_ min^−1^ (g dry weight)^−1^ in KH; *P* < 0.05]. In addition, there is some indication that the relationship between heart rate and $M{\dot{V}}_{O_{2}}$ shows two phases; below 525 beats min^−1^ $M{\dot{V}}_{O_{2}}$ appears independent of heart rate, whereas above 525 beats min^−1^ $M{\dot{V}}_{O_{2}}$ declines gradually as heart rate increases (Fig. 3 *C*).

The KH_metab_‐perfused hearts showed an apparent positive correlation between RPP and myocardial oxygen consumption (Fig. 3 *D*), again showing some evidence of a two‐phase response. However, closer inspection reveals that this RPP–$M{\dot{V}}_{O_{2}}$ relationship is, in fact, ‘upside‐down’; that is, as both LVDP and $M{\dot{V}}_{O_{2}}$ decline with increasing heart rate, high rates are associated with a low RPP and a low $M{\dot{V}}_{O_{2}}$ and, conversely, low heart rates with a high RPP and high $M{\dot{V}}_{O_{2}}$. Figure 3 *E* shows the relationship between heart rate and lactate production. This relationship again shows two phases, again with an inflection at ∼525 beats min^−1^, above which increases in heart rate appear to be linearly associated with increased lactate production.

### Effects of β‐adrenoceptor stimulation on RPP and $M{\dot{V}}_{O_{2}}$

**Figure 4:**
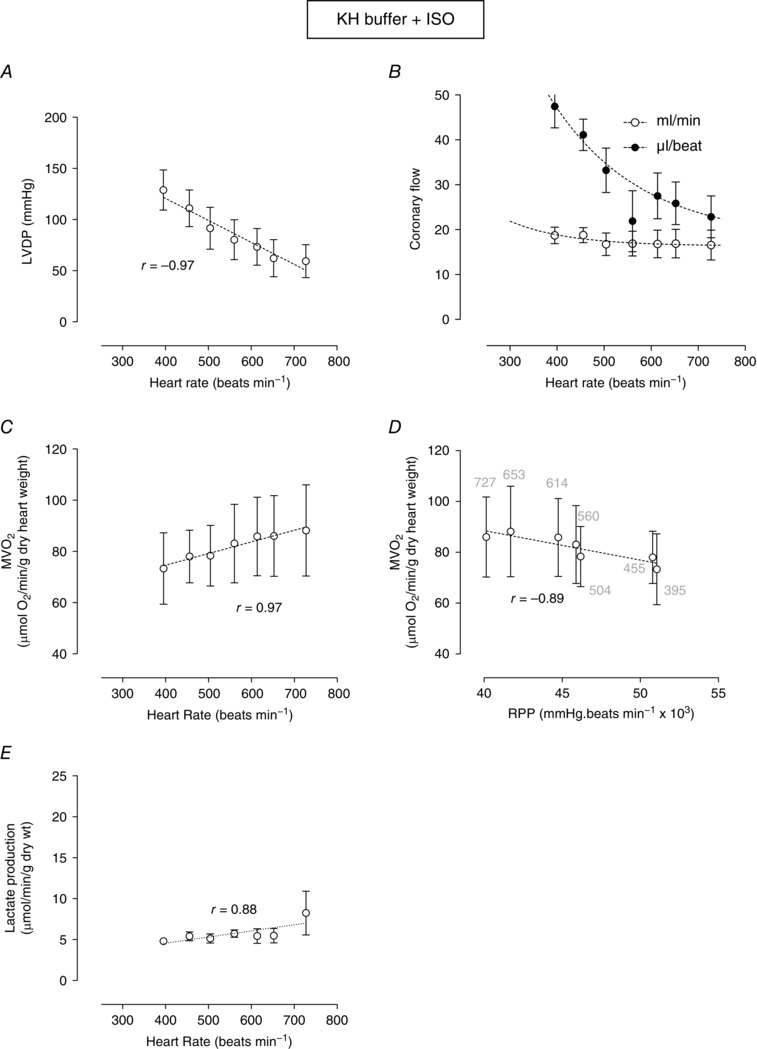
**Effect of isoprenaline (ISO; 10 nm) on** $M{\dot{V}}_{O_{2}}$ **, LVDP, coronary flow and lactate production plotted as a function of heart rate or RPP in hearts perfused with KH buffer** Relationship between pacing rate and LVDP (*A*) and coronary flow (*B*). Myocardial oxygen consumption is plotted as a function of heart rate (*C*) or RPP (*D*). The lactate production measured in coronary effluent at each pacing rate is shown in *E*. Data are means ± SEM (*n* = 5). The *r* value is the Pearson correlation for each linear relationship. Non‐linear fits are as described in the Methods.

Figure 4 *A* shows that, although ISO (10 nm) had the expected positive inotropic effect at all heart rates, the LVDP–frequency relationship remained negative (−0.97 mmHg beat min^−1^) (see also Fig. S1). Isoprenaline at baseline (400 beats min^−1^) caused a 52% increase in coronary flow (18.7 ± 1.8 ml min^−1^) compared with KH alone (12.3 ± 0.7 ml min^−1^). Further increase in heart rate in the presence of ISO caused very little change in absolute coronary flow and, as previously observed, a substantial decline in flow per beat (Fig. 4 *B*). These changes in coronary perfusion were accompanied by increased oxygen demand (Fig. 4 *C*; *r* = 0.97). The relationship between RPP and $M{\dot{V}}_{O_{2}}$ is shown in Fig. 4 *D*. This relationship, which was absent in KH buffer alone (Fig. 1 *C*), was transformed into a significant negative correlation by β‐adrenoceptor stimulation. This appears to be associated with a gradually developing demand‐induced ischaemia (Fig. 4 *B*) as indicated by a significant increase in lactate production (Fig. 4 *E*).

**Figure 5:**
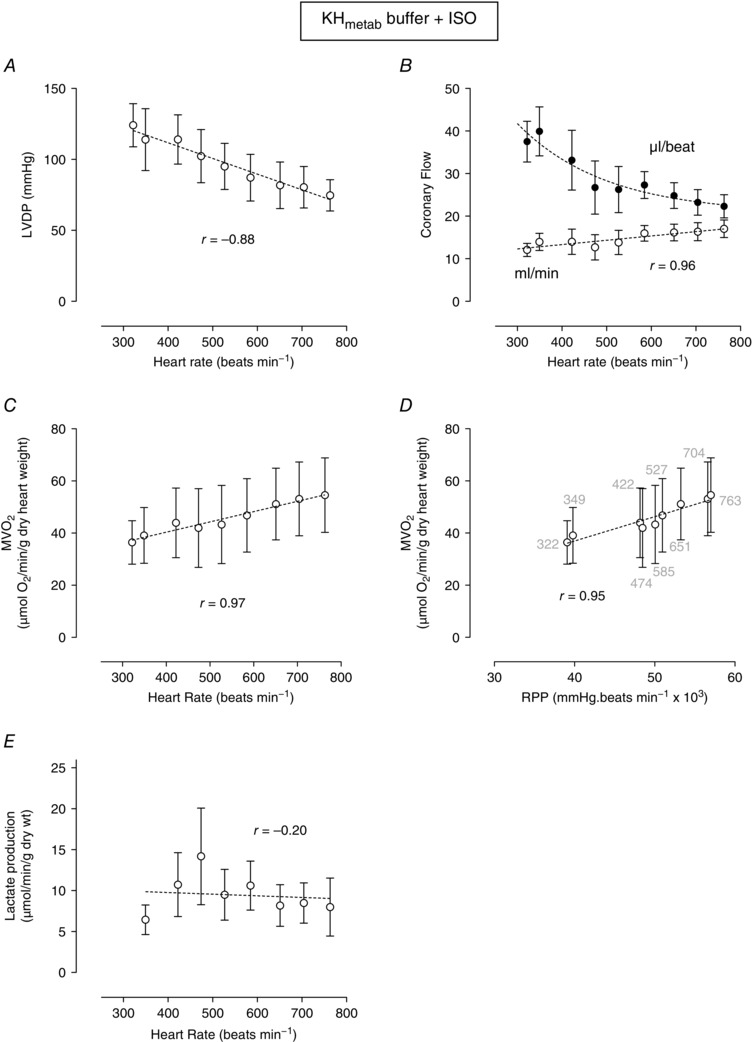
**Effect of ISO (10 nm) on** $M{\dot{V}}_{O_{2}}$ **, LVDP, coronary flow and lactate production plotted as a function of heart rate or RPP in hearts perfused with KH_metab_ buffer** Relationship between pacing rate and LVDP (*A*) and coronary flow (*B*). Myocardial oxygen consumption is plotted as a function of heart rate (*C*) or RPP (*D*). The lactate production measured in coronary effluent at each pacing rate is shown in *E*. Data are means ± SEM (*n* = 5). The *r* value is the Pearson correlation for each linear relationship. Non‐linear fits are as described in the Methods.

Figure 5 shows the data from hearts perfused with KH_metab_ buffer in the presence of β‐adrenergic stimulation. In contrast to the KH_metab_ buffer results in the absence of ISO (Fig. 3 *B*), as heart rate increased so did coronary flow (Fig. 4 *B*). This increase in coronary flow resulted in increased oxygen extraction and increased oxygen consumption (Fig. 5 *C*). The relationship between RPP and $M{\dot{V}}_{O_{2}}$ is shown in Fig. 5 *D*. This relationship, which was ‘upside‐down’ in KH_metab_ buffer (Fig. 3 *D*), was transformed into a significant positive correlation by ISO stimulation (Fig. 5 *D*). Coronary vasodilatation caused by ISO resulted in reduced lactate production (Fig. 5 *E*).

### Effect of ISO‐mediated increase in heart rate on the RPP–$M{\dot{V}}_{O_{2}}$ relationship

**Figure 6:**
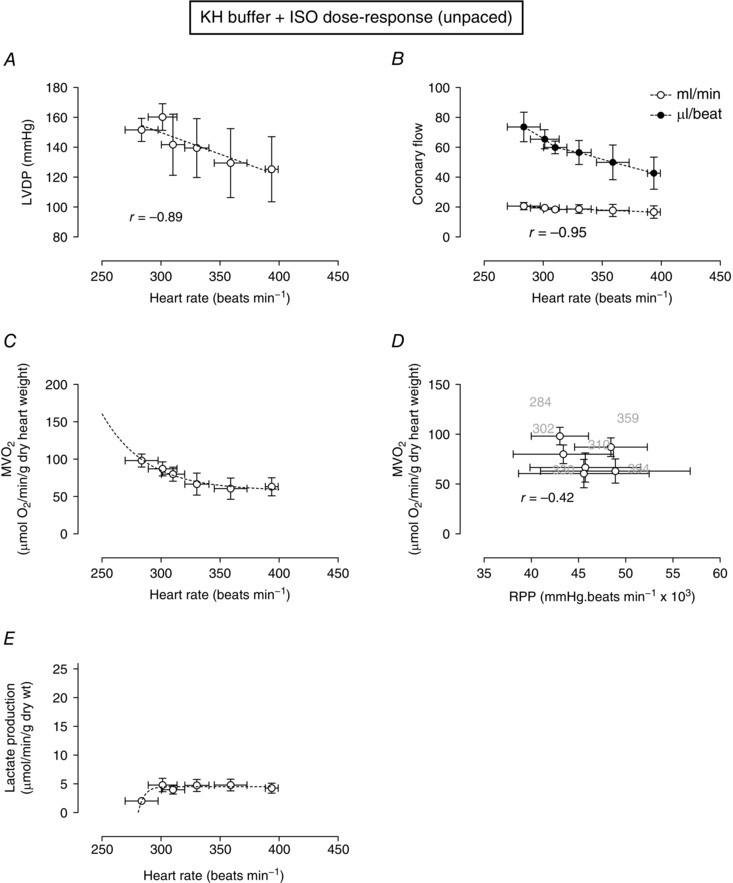
**The effect of changing heart rate with increasing concentrations of ISO on LVDP, coronary flow, oxygen consumption and lactate production** Hearts were unpaced, and ISO concentration was increased stepwise every 10 min. The ISO concentrations used were 0.01, 0.1, 1, 10, 100 and 1000 nm. Relationship between heart rate and LVDP, *A* and coronary flow, *B*. Myocardial oxygen consumption is plotted as a function of heart rate, *C* or RPP, *D*. The lactate production measured in coronary effluent at each ISO concentration is shown in *E*. Data are means ± SEM (*n* = 5). The *r* value is the Pearson correlation for each linear relationship. Non‐linear fits are as described in the Methods.

Given that electrical pacing is not the usual mechanism for increasing rate *in vivo* (even in the presence of low‐dose β‐receptor stimulation, as was done in Figs 4 and 5), in these experiments rate was increased in unpaced hearts by constructing a cumulative ISO dose–response curve. Figure 6 *A* shows the relationship between LVDP and heart rate at various concentrations of ISO. Even in the presence of increasing ISO concentrations, the force–frequency response remained negative. At lower ISO concentrations (heart rates), absolute coronary flow and flow per beat were substantially higher than in the absence of ISO but similar to that in 10 nm ISO (Fig. 6 *B*). As heart rate was increased (with increasing ISO concentrations), absolute coronary flow remained relatively constant, whereas flow per beat declined. Oxygen consumption also declined as heart rate increased (Fig. 6 *C*). Again, as in the KH buffer, for KH buffer + ISO, there was no correlation between RPP and $M{\dot{V}}_{O_{2}}$ (Fig. 6 *D*) and no increase in lactate production (Fig. 6 *E*).

## Discussion

This study demonstrates that, despite its widespread use, RPP is an unreliable index of $M{\dot{V}}_{O_{2}}$ or cardiac ‘effort’. Although the provision of substrates or β‐adrenergic stimulation changed the shape of the RPP–$M{\dot{V}}_{O_{2}}$ relationship, neither intervention resulted in a clear positive correlation between RPP and oxygen consumption. In KH buffer alone, although $M{\dot{V}}_{O_{2}}$ increased with heart rate, LVDP declined. Hence, the product of HR × pressure (RPP) remained flat.

In isolated papillary muscle or trabecular preparations, the FFR is often studied at relatively unphysiological low rates of electrical stimulation, high Ca^2+^ concentrations, in the absence of catecholamines or vagal tone and, not infrequently, at room temperature (for review, see Bers, 2000). In these conditions, the FFR is clearly species dependent and has a positive slope in guinea‐pig, rabbit, dog and human cardiac muscle and is flat or negative in rat and mouse cardiac muscle (Schouten & ter Keurs, 1986; Redel *et al*. 2002). In most species, the increase in inotropy with increasing rate reflects an increase in the time‐averaged Ca^2+^ influx, a decrease in the diastolic interval available for Ca^2+^ extrusion, a rise in intracellular Na^+^, and a consequent rise in sarcoplasmic reticulum (SR) Ca^2+^ load and fractional Ca^2+^ release (for review, see Bers, 2000). In rat and mouse cardiac muscle at low rates of stimulation, the SR is relatively Ca^2+^ loaded, intracellular Na^+^ is already elevated, and rate increases may even unload the SR of Ca^2+^. This, combined with the refractoriness of the SR Ca^2+^ release process, may account for the negative inotropy on increasing stimulation rates in these species (Bers, 2000).

In isolated muscles, the failure to study the FFR at higher rates of stimulation and at 37°C is, in part, driven by concerns about the adequacy of oxygen and substrate delivery in these superfused preparations (Schouten & ter Keurs, 1986). Although it seems unlikely that muscle bundles of similar sizes from species with a positive FFR are somehow better oxygenated than those from rats, concern remains that an element of the negative FFR at high pacing rates may reflect inadequate oxygenation and/or substrate delivery. Indeed, in a study by Layland & Kentish (1999), when thin (80–250 μm) trabeculae were perfused at 37°C in 1 mm Ca^2+^, the FFR remained positive over the range 0–12 Hz. In subsequent studies, Raman *et al*. (2006) also showed that smaller muscle diameters (<150 μm) are correlated with improved muscle performance, particularly at high pacing rates. Whether these observations reflect the inadequacy of diffusion to maintain viability of larger muscles or an intrinsic difference in the cellular properties of smaller muscles has not been determined.

In isolated hearts, as in isolated muscles, the developed pressure–frequency relationship is typically positive (or biphasic) in most larger species and is negative or flat in rats or mice (Taylor & Cerny, 1976; Mattheussen *et al*. 1996; Maier *et al*. 1997; Sutherland *et al*. 2003). In the present study, although arterial perfusion might improve oxygen delivery, it was important to rule out the possibility that a negative FFR at higher pacing rates simply reflects inadequate perfusion and demand ischaemia (see (Kuzmiak‐Glancy *et al*. 2015). Krebs–Henseleit buffer‐perfused hearts have very high coronary flows (10–20 ml min^−1^ g^−1^) compared with those measured *in vivo* or in blood‐perfused hearts (∼1 ml min^−1^ g^−1^). These high coronary flows may limit locally derived vasoactive agents from achieving concentrations necessary to cause appropriate or sufficient dilatation in response to increases in heart rate. However, despite this, in the present study, in normal KH‐perfused hearts, the PCr/ATP ratio was not reduced at higher pacing rates. This suggests that the decline in LVDP was not attributable to progressive demand ischaemia. In isolated hearts supplied only with glucose and no insulin, it is also reasonable to consider the possibility that demand ischaemia may also be induced by inadequate substrate delivery as well as inadequate O_2_ delivery. However, the lack of significant frequency‐dependent lactate production and the finding that the provision of alternative substrates did not affect the negative FFR, or indeed the RPP–$M{\dot{V}}_{O_{2}}$ relationship, suggests that substrate delivery is not the limiting factor in this context.

*In vivo*, heart rate is never substantially elevated in the absence of β‐adrenoceptor occupation. Vagal withdrawal may modulate heart rate, but substantial increases in rate during exercise are always accompanied by sympathetic outflow and β‐adrenergic stimulation. On a cellular level, β‐adrenergic stimulation will phosphorylate key proteins involved in excitation–contraction coupling, such as L‐type Ca^2+^ channels, ryanodine receptors, phospholamban and phospholemman. These latter two protein kinase A substrates may act in concert to limit cytosolic Ca^2+^ and Na^+^ accumulation when heart rate increases *in vivo*, but this effect may be absent when isolated preparations are electrically paced in the absence of β‐adrenergic stimulation. This raises the question: does the negative FFR in rats and mice ever occur *in vivo*? In fact, it seems teleologically surprising that, in a fight‐or‐flight situation, the contractility of the rat or mouse heart would decline when heart rate goes up. The answer to this question is beyond the scope of the present study, but it is interesting to note that the negative FFR of an isolated mouse heart could be transformed into a positive FFR if heart rate was raised by β‐adrenergic stimulation rather than by electrical pacing (Kennington, 2009). In the present study (Fig. 6), the rat heart, unlike the mouse heart, maintained a negative FFR even when heart rate was raised by cumulative increases in isoprenaline concentration, suggesting that the negative FFR of the rat heart is not the result of a lack of β‐adrenergic stimulation.

As discussed above, the lack of correlation between RPP and $M{\dot{V}}_{O_{2}}$ may simply be a limitation of *ex vivo* perfusion and the inadequacies of oxygen delivery, receptor stimulation or substrate provision, rather than a species‐specific phenomenon. We have been unable to find studies in the literature relating these variables in Langendorff‐perfused hearts from other small animal species (i.e. rabbit, guinea‐pig etc.), in which the FFR is reported to be positive. Likewise, no studies have measured $M{\dot{V}}_{O_{2}}$ and the FFR *in vivo* in rats or mice, although, as discussed above, it would be teleologically surprising if the negative FFR persists *in vivo*. It would be interesting to repeat the present study not only in small animals *in vivo* but also in KH‐perfused rabbit hearts and hearts of both species perfused with erythrocytes or perfluorocarbons to improve O_2_ delivery.

When designing the present study, the assumption was made that hearts perfused with the enhanced substrate mix (KH_metab_ buffer) would be more stable and less prone to demand ischaemia than glucose‐fed hearts (KH buffer). Surprisingly, this was not borne out by the results. In KH_metab_ buffer, LVDP and coronary flow were consistently lower than in KH buffer and, on increasing heart rate, KH_metab_‐perfused hearts produced more lactate. The inclusion of BSA in the perfusion solutions increased both the viscosity and the oncotic pressure of these solutions. Whether it is a change in viscosity or some effect on the tissue oedema (known to accompany crystalloid perfusion), there is demonstrably a reduction in coronary flow in hearts perfused with the KH_metab_ buffer. If coronary flow is reduced for reasons of viscosity and/or changes in oedema, then as the inclusion of BSA does not alter or improve the oxygen‐carrying capacity, this may limit oxygen delivery and may explain why these KH_metab_‐perfused hearts are more susceptible to demand ischaemia.

**Figure 7:**
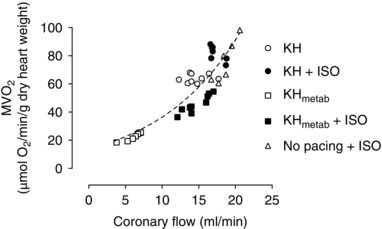
**Relationship between oxygen consumption and coronary flow for all hearts** Data are replotted from the data in Figs 1, 3, 4, 5 and 6. The curve is an exponential fitted using the following equation: *Y* = 0.342 × exp(0.1205*X*).

Finally, it is interesting to note that in the crystalloid‐perfused rat heart it is coronary flow (i.e. oxygen delivery) and not heart rate that is best correlated with oxygen consumption. The correlation between $M{\dot{V}}_{O_{2}}$ and heart rate was poor across all protocols; sometimes being positive, sometimes flat and sometimes negative. In contrast, there was a good correlation in all conditions studied between $M{\dot{V}}_{O_{2}}$ and coronary flow (oxygen delivery) (Fig. 7). The correlation between oxygen delivery and oxygen consumption was positive, non‐linear and increased by β‐adrenergic stimulation in all protocols. This observation was made more than 50 years ago in an elegant series of studies published in 1963 showing, in non‐working isolated heart preparations, that increasing coronary flow increases oxygen consumption in isolated non‐working hearts when demand is constant (Kahler *et al*. 1963). That is, under these conditions, it is delivery of oxygen that determines $M{\dot{V}}_{O_{2}}$ rather than the increase in O_2_ consumption that drives changes in coronary flow. In the present study, both contractility (i.e. demand) and coronary flow (i.e. O_2_ delivery) change with changes in pacing rate and hence while $M{\dot{V}}_{O_{2}}$ and coronary flow correlate, this correlation may be a undefined mixture of cause and effect.

In conclusion, although many studies use RPP in the isolated Langendorff‐perfused rat heart, the negative FFR in this preparation means that RPP is an unreliable index of oxygen consumption or ‘cardiac effort’ and should be avoided.

## Additional information

### Competing interests

None declared.

### Author contributions

All experiments were conducted in the laboratories of the Cardiovascular Division, King's College London. D.A.: experimental design, heart perfusions, lactate analysis, nuclear magnetic resonance spectroscopy, primary data analysis and manuscript preparation. H.R.L.: heart perfusions, lactate analysis and data analysis. M.J.S.: principal investigator and grant holder, study hypothesis, experimental design, secondary data analysis and manuscript preparation. All authors have approved the final version of the manuscript and agree to be accountable for all aspects of the work. All persons designated as authors qualify for authorship, and all those who qualify for authorship are listed.

### Funding

This work was supported by a British Heart Foundation Programme Grant (RG/12/4/29426) to M.J.S. and a British Heart Foundation 4 year MRes/PhD studentship (FS/13/55/30643) to H.R.L.

## Supporting information


**Table S1**: Absolute values of arterial and venous O_2_ content, A‐V oxygen consumption and coronary flow at different heart rates in KH perfused hearts. These data were used to generate the relationships shown in Figure 1. Date are mean ± SEM (n=8/group).
**Figure S1**: Comparison of the effects of isoprenaline (ISO) on the relationship between pacing rate and left ventricular developed pressure (LVDP) in hearts perfused with KH (A) or KH_metab_ (B). Data are re‐plotted from Figures 1A, 3A, 4A and 5A.
