# Supplementary material for: Is rate–pressure product of any use in the isolated rat heart? Assessing cardiac ‘effort’ and oxygen consumption in the Langendorff‐perfused heart
Source: Exp Physiol. 2015 Dec 16;101(2):282–94. doi: 10.1113/EP085380 (PMC4833194; doi:10.1113/EP085380)
Supplement: Supplementary file 1 — Table S1: Absolute values of arterial and venous O2 content, A‐V oxygen consumption and coronary flow at different heart rates in KH perfused hearts. These data were used to generate the relationships shown in Figure 1. Date are mean ± SEM (n=8/group). Figure S1: Comparison of the effects of isoprenaline (ISO) on the relationship between pacing rate and left ventricular developed pressure (LVDP) in hearts perfused with KH (A) or KHmetab (B). Data are re‐plotted from Figures 1A, 3A, 4A and 5A. [file EPH-101-282-s001.doc]

Data Supplement

Dunja Aksentijević, Hannah R. Lewis, Michael J. Shattock: **Is rate-pressure product of any use in the isolated rat heart?** Assessing cardiac ‘effort’ and oxygen consumption in the Langendorff-perfused heart

| | Heart rate | Coronary flow | Arterial O_2_ | Venous O_2_ | Absolute A-V difference | O_2_ consumption | | --- | --- | --- | --- | --- | --- | | (bpm) | (ml/min) | (µmoles/ml) | (µmoles/ml) | (µmoles/ml) | (µmoles/min/g) | | 326 | 12±1 | 1.33±0.18 | 0.34±0.05 | 0.99±0.16 | 63.0±7.1 | | 373 | 13±1 | 1.15±0.09 | 0.33±0.04 | 0.82±0.09 | 60.5±7.8 | | 434 | 14±1 | 1.15±0.08 | 0.33±0.07 | 0.82±0.09 | 61.8±8.4 | | 476 | 14±1 | 1.16±0.09 | 0.26±0.04 | 0.91±0.08 | 68.0±7.1 | | 527 | 14±1 | 1.12±0.10 | 0.25±0.04 | 0.88±0.08 | 67.3±7.5 | | 589 | 15±1 | 1.00±0.12 | 0.27±0.04 | 0.73±0.09 | 60.1±8.9 | | 631 | 15±1 | 0.99±0.09 | 0.23±0.04 | 0.76±0.09 | 63.9±8.2 | | 693 | 16±1 | 0.95±0.09 | 0.21±0.04 | 0.74±0.07 | 67.3±8.0 | | 738 | 18±1 | 0.90±0.11 | 0.27±0.04 | 0.64±0.08 | 63.6±9.6 | |
| --- | --- | --- | --- | --- | --- | --- | --- | --- | --- | --- | --- | --- | --- | --- | --- | --- | --- | --- | --- | --- | --- | --- | --- | --- | --- | --- | --- | --- | --- | --- | --- | --- | --- | --- | --- | --- | --- | --- | --- | --- | --- | --- | --- | --- | --- | --- | --- | --- | --- | --- | --- | --- | --- | --- | --- | --- | --- | --- | --- | --- | --- | --- | --- | --- | --- | --- |

***Table S1:*** Absolute values of arterial and venous O_2_ content, A-V oxygen consumption and coronary flow at different heart rates in KH perfused hearts. These data were used to generate the relationships shown in Figure 1. Date are mean ± SEM (n=8/group).

***Figure 1S:*** Comparison of the effects of isoprenaline (ISO) on the relationship between pacing rate and left ventricular developed pressure (LVDP) in hearts perfused with KH (**A**) or KH_metab_ (**B**). Data are re-plotted from Figures 1A, 3A, 4A and 5A. **Note:** in KH perfused hearts ISO (10nM) has little sustained effect on LVDP. This relatively low concentration of ISO only results in a sustained increase in LVDP by about 10mmHg at all heart rates. The original recordings show that ISO caused a much larger but transient inotropy which peaked, but then waned, such that the steady-state inotropy after 10 mins was only +10mmHg. The response to ISO was significantly different in KH_metab_-perfused hearts (See Panel B). Not only was the inotropy more sustained (ie did not wane), unlike KH alone (where the regression lines are parallel) in KH_metab_ the lines diverge at higher rates suggesting that at higher rates KH_metab_ allows for a relatively greater inotropy than KH alone. This is likely to be attributable to the much higher coronary flows in the KH_metab_ ISO group and reaffirms the conclusion at the end of the paper that coronary flow is an important determinant of cardiac efficiency.
